# Morbid Effects of Digitalis in a Nervous Subject

**Published:** 1831

**Authors:** 


### XXVII.

#### Morbid Effects of Digitalis in a Nervous Subject. By M. Sabatier.

[Hebdomadaire.]

Case. Mad. M. aged 23 years, of acute sensibility, amiable temper, was born in affluence, but doomed to misfortunes. She married, but lost her first child, on whom she doated. This was followed by other moral ills which we need not recapitulate. In March 1830, she experienced, for the first time, an attack of epilepsy, which was repeated twice afterwards, in the course of four or five months. Ligatures on the extremities warded off several threatened attacks. But extreme nervous susceptibility, palpitations, &c. which had been felt for some years, became now very much exasperated. The appetite diminished, the digestion was weak, and all mental or corporeal exertion became distressing. Such was the history previously to May 1831, at which epoch the palpitations assumed a more serious character than ever. The pulse was very frequent and extremely small. The right eye was the seat of most dolorous sensations, and numerous electrical sparks. The attacks of palpitation came on without any exertion or mental emotion. Auscultation did not detect any enlargement of the heart. On the 17th May, small doses of infusion of digitalis were prescribed, and for two days the lady seemed better; but on the third day a train of symptoms occurred, which proved very alarming. Great weakness of the limbs, sentiment of constriction under the sternum, vertigo, faintings, palpitation, slowness of pulse, with much irregularity, sense of coldness and numbness of the extremities, difficulty of breathing, &c. After the exhibition of æther and some other medicines, a train of the most anomalous and nervous symptoms succeeded, which fill whole pages of the French journal from which we quote, but which need not here be enumerated. Among these symptoms, however, we may state that an obstinate spasmodic constriction of the œsophagus was one of the most prominent and distressing. These phenomena gradually subsided, and have afforded the imaginative Frenchman an opportunity of throwing off a series of conclusions and deductions, no less than *nine* in number, and of the most erudite and hypothetical nature. These we shall leave where we found them. The quantity of digitalis is not very accurately defined; but the effects were of a very embarrassing kind while they lasted.

---

